# Supplementary material for: Patterns of Intron Gain and Loss in Fungi
Source: PLoS Biol. 2004 Nov 30;2(12):e422. doi: 10.1371/journal.pbio.0020422 (PMC532390; doi:10.1371/journal.pbio.0020422)
Supplement: Table S1 — Also available at http://genes.mit.edu/NielsenEtAl/. (4.3 MB ZIP). [file pbio.0020422.st001.zip › NielsenEtAl/html/151.html]

AN0929.1.NCU03176.1.MG03007.1.FG09539.1


```
 CLUSTAL W (1.82) Multiple Sequence Alignments - Introns Inserted


Sequence 1: NCU03176.1	654 aa
Sequence 2: MG03007.1	831 aa
Sequence 3: FG09539.1	790 aa
Sequence 4: AN0929.1	728 aa
Alignment Length: 889 aa
Number Identitical Residues: 204 aa
Alignment Score (without introns) 11715


MG03007.1 	MSSHPSPGLG-----------QQPLKSALKTDDEAAERSPAVKA1VQI~AEPEMTKIEPA
NCU03176.1	--------------------------------------------~---~-----------
FG09539.1 	MSAEPSPATNGSPAQQQQQPQPPPLRSALKSEEDIERPLPPSGS~LKV1QIAEPESEIQT
AN0929.1  	----------------------------MDSDKSAKG-----HY~LGV~ETRLPSAQNPS
          	                             .:...                         :

MG03007.1 	QQAPGSPEDGFRKQFTAGLAKRLS-GRLPAGSSRSSLMSN-ASGDAPNSTASSTDSTQQI
NCU03176.1	-------------------------------------MDE-SPAHGP-------------
FG09539.1 	LEDDSQPTKQFHTNLRRRLSGKLPPSQLPNSSSRSSLSDSPSTSRAPEEPAPTSN-----
AN0929.1  	QDSAATSEANTTRQRRVSVSKVTVRQSFHYPTSAGALNHQVPNANESVTKED--------
          	 .  . .      .     :           :: .:   . . .  .             

MG03007.1 	AEEKHHRHHHHFHLRHRSNSIEHQQQQQQQQQQQQGGHVDSRLVMQVLQWIGREKEKKES
NCU03176.1	------------HGRHR-NKIDR---------------ISERLVAQVAEWIQHEKTKRET
FG09539.1 	------------PSSHHHHSHRR-------------QYYSEKLLAQVGDWLEHERAKAHA
AN0929.1  	------------IDRWLKQSEEE---------------SRHNLFSQVYEWLSREKSKRKH
          	                  :.  .                  .*. ** :*: :*: * . 

MG03007.1 	RRKRRMTRHRKSKSPPSTDDAS----------------GMQASPSRPRADSIDSDGSDVS
NCU03176.1	RKTGKLLRRRK--TPPIHVNGE----------------PTVSSPTRLRSDSVSSDSSEVS
FG09539.1 	RKSKKTSRRRKSKSPNKENQSKSTGESEPATASTSAQGSEQSNNGRPRSDSVDSDSSDTS
AN0929.1  	SNKSNGSTNGSGNGFDGEGDAHG------------------DGAQLTRTVSQGSD-TTLA
          	 .. .   . ...      :. .                   .    *: * .** :  :

MG03007.1 	LDQLQKIVDEGLAAMGIDPATAASATNPPRVNPRRRLSKHKSRSSLQLY--RAVSSDTDY
NCU03176.1	LDRLQKILNDGMSALGLD--------QVPQLGPR--LGRRRSRRSLKAPPARTASSDTEF
FG09539.1 	FDRLQRILEESLAHMGLN--------GLPHMAPK--MARPKQRKVPSRSSLLGPSSDTEY
AN0929.1  	LDALEKILLQYASRYDSR--------TMSTCSSR----RSTRRRHPIKGLRRGSVSESDY
          	:* *::*: :  :  .            .   .:    :   *            *::::

MG03007.1 	VDGDVLVPSCDAVLDNSKTLSFSGGKAASTEDLAAAQSRREEKERQAWSTFKGEILRLAH
NCU03176.1	FDGDVVVPSCDVFLDNSKTMSYSGGKAT-TDDTPSQASRREEKEKQAWLTFKNEIIRLAH
FG09539.1 	ADGDAIVPSCDVWLDNTKTMSYAGGNADAEDQDINDTDIKADKGKECWLSFKNEIIRIAH
AN0929.1  	-ELDPITPAVDAVLDNSRTLSYTGGGVESEDGDAASITR--AKDREAWVVFKSELLRLTH
          	 : * :.*: *. ***::*:*::** . : :           * ::.*  **.*::*::*

MG03007.1 	TLKLKGWRRVPLECGDGLAVERLSGALTNAVYVVSPPTEIVSNMSID-DVSKKPKKPPP~
NCU03176.1	TLRLKGWRRIPLNGGETISVERLSGALTNAVYVVSPPPESILPP-------QEGKRQPE~
FG09539.1 	TLRLKGWRRIPLGGGDSISVVRLSGALTNAVYVVTPPKEIDETD---------GKRKPT~
AN0929.1  	TLQLKGWRKIPMELASEIDVVRLSGALTNAVYKITPPQNIPPPKAEDGSYTLVPRRPPP2
          	**:*****::*:  .. : * *********** ::** :     : ... :   :: *  

MG03007.1 	KLLLRIYGPQVEQLIDREKELGVLKRLARKKIGPRMLGTFTNGRFEQYLNATTLTAANLR
NCU03176.1	KVLLRVYGPQVEHLIDREIELGVLKRLARKKIGPRLLGTFLNGRFEQYFNSTTLTPENLR
FG09539.1 	KVLLRIYGPQVEHLIDRDNELSVLQRLARKKIGPRLLGTFQNGRFEQYFESITLTPMDLR
AN0929.1  	KLLLRIYGPQVDHLIDREKELQILRRLGRKNIGPKVLGTFNNGRFEEYFEARPLTPKELR
          	*:***:*****::****: ** :*:**.**:***::**** *****:*::: .**. :**

MG03007.1 	EPETSKMIAKRMKELHVGVELLESELAAGPNVWVNWDSWLDAVERTVLALD----KKVLE
NCU03176.1	EPETSKQIAKRMRELHDGVELLEHEKDEGPGVWRNWDRWLDQAEKTAMYLDSQVAAQLQE
FG09539.1 	DPDTSRSIAKRMRELHEGIDLLPHEREGGPATWKSWDQWLDNVERIATYLDNEYEKHVEG
AN0929.1  	DPSTMKQIAKRMRELHDGIDLLDEERENGPMVFHNWDKWVDRCEQVISWVDAEIKSPLNE
          	:*.* : *****:*** *::**  *   ** .: .** *:*  *:    :* .    :  

MG03007.1 	SPAD----AKDWRKGGFVCGVEWHVFKGLVERYRVHLTEHH-GGQKKIREKL~VFAHND0
NCU03176.1	STRH----KETWKTRGFVCGVEWPVFRKMVQKYRKFLEDYY-GSPTKIREKL0------~
FG09539.1 	QHGQQDSVAHAWKSRGYVCGTTWPEFRNMMTKYRVHLNSFYKGGQREVKDSL~VFAHSD0
AN0929.1  	GKAA----SEPWRRRGYVCGVPWPKFRKAVEDYRKWLIVDS-GGVHEIKRQL~VFGHND0
          	         . *:  *:***. *  *:  :  **  *     *.  ::: .*   . .. 

MG03007.1 	TQYGNILRIRPDDEKSPLLQPANEHKQLVVIDFEYAAANTPGLEFANHF~TEWTYNYHDA
NCU03176.1	TQYGNILRIRPDDKKSPLLQPANEHKQLIVIDFEYAGANLAGLEFANHF~SEWTYNYHDP
FG09539.1 	TQYGNILRIRPDDEKSPLLQAANKHKQLIVIDFEYAGPNTRGLEFANHF~NEWTYNYHDA
AN0929.1  	TQYGNLLRMEPS-QQSPLLLPQNEHKQLVVIDFEYASANTPGLEFANHF0TEWCYNYHDA
          	*****:**:.*. ::**** . *:****:*******..*  ******** .** *****.

MG03007.1 	VAPHACNASRYPTPEQQRRFVRAYVDHS--------HPRRP--SSSSSS--SSLAQTP--
NCU03176.1	VTPHVCDAAKYPTLEQQRRFIKAYVDHQP------KFPSVPGVSTPSVT--PSSAETPRP
FG09539.1 	AAPWACDVRRYPTPDEQRRFIKAYVDHRPRFQGSNSTPRLAPVDSNASSGMSTPLATP--
AN0929.1  	EKPWACNNQLYPTPEQQHQFVTAYLTHR---------PGLGSRVSPSITPIMRPLSAS--
          	  * .*:   *** ::*::*: **: *          *      : : :       :.  

MG03007.1 	-TMNATTSTSSIVDFMLDARVPPGGWKEEETRRDAESDIKIRALLEETRLWRPANSAQWV
NCU03176.1	GMLQSTNSASSIVEFMLDARVPPGGWKEEDARREAESEKRIKELLEETRLWRVANSAQWV
FG09539.1 	-GNPAANSSSSIVEFMLDARVPPGGWSAAERANDEQRDQRVRELLEETRQWRPANHAHWI
AN0929.1  	-TPTMTPLDLNATSPDLAPQRPP---DNIERSAQDTLEAETQFLIRQTRLWRVLNSAQWV
          	     :    . ..  * .: **   .  :   :   : . : *:.:** **  * *:*:

MG03007.1 	AWGVVQAKLPAGVAREVEAAKKVDEAKKAEEAKLNNDNIDEQERPKTEKEQEAEAVAEAA
NCU03176.1	VWGIIQAKIPG----------------------LNDGAKDVVTAP-------AGSVPDEE
FG09539.1 	AWGLVQAKIPG----------------------LDESIKE--------------------
AN0929.1  	AWGIVQAKVPG----------------------MEDDGSSTPTPP---------------
          	.**::***:*.                      :::.  .    .               

MG03007.1 	AINEEEAVADEFDYLGYARERAMFFLGDC~VLMGLIKLEDLPEATRDKVK--FVDY
NCU03176.1	--LEAEEEADAFDYLGYSQERAFFFWADC0SHVTARRLR----AMRGR--------
FG09539.1 	---EDLLSPDEFDYLSYAQDRTMFFWGDC~VRMGLVKLEQLPPKLQQQVK--TAEY
AN0929.1  	-VDSDVDESDEFDYLAYAQDRAFFFWADL~VALGFVQKEQLPESLAEVVDGRILEY
          	   .    .* ****.*:::*::** .*    :   : .. .       ..   .
```
